# Complete Genome Sequence of Seoul Virus Strain Tchoupitoulas

**DOI:** 10.1128/genomeA.00480-16

**Published:** 2016-06-09

**Authors:** Rory W. Miles, Kuiama Lewandowski, Barry Atkinson, Steven T. Pullan, Graham Lloyd, Daniel Bailey

**Affiliations:** National Infection Service, Public Health England, Porton Down, United Kingdom

## Abstract

Seoul virus (genus *Hantavirus*; family *Bunyaviridae*) is an emerging pathogen associated with cases of acute kidney injury in several countries across the globe. We report here the whole-genome sequence of the Tchoupitoulas strain of Seoul virus isolated in New Orleans, LA.

## GENOME ANNOUNCEMENT

Hantaviruses are members of the *Bunyaviridae* family; as such, they are enveloped negative-sense single-stranded RNA viruses with a trisegmented genome. The large (L) segment encodes a viral RNA polymerase, the medium (M) segment encodes a glycoprotein precursor, and the small (S) segment encodes the nucleocapsid protein (1). Seoul virus (SEOV) is a globally spread hantavirus linked with the worldwide presence of its reservoir, *Rattus norvegicus* (brown rat) (2). SEOV presents a significant public health risk as a cause of hemorrhagic fever with renal syndrome, which is associated with acute kidney injury (AKI) (2). Six cases of hantavirus-associated AKI were diagnosed in the United Kingdom between 2012 and 2014 (3). Within Europe, SEOV has recently been isolated from brown rats in Yorkshire, Wales, and Sweden, and human SEOV infections were identified in Wales and France (4 – 7). Despite their worldwide spread, SEOV strains are closely related and have low genetic diversity (8). SEOV strain Tchoupitoulas (TCH) was isolated from the pancreas of a brown rat trapped near the Mississippi River in New Orleans, LA (9). Currently, only the S segment sequence has been published (10). Here, we report the first full-genome sequence of TCH.

The virus was isolated in 1984, propagated in Vero E6 cells by the staff of the Rare and Imported Pathogens Laboratory in 1992, and deposited into the National Collection of Pathogenic Viruses in 2001, under catalog no. 0107221v. Viral RNA was extracted using the QIAamp viral RNA minikit (Qiagen), following the addition of 10 ng/µl GenElute-linear polyacrylamide (Sigma-Aldrich). The sample was digested with Turbo DNase I (Thermo Fisher Scientific) and purified using the RNA Clean & Concentrator kit (Zymo). Single-primer isothermal linear amplification (SPIA) cDNA was prepared from total RNA using the Ovation RNA-seq version 2 system (NuGen). One and a half nanograms of SPIA cDNA was prepared for sequencing, according to the Illumina Nextera XT protocol, and the 2 × 150-bp paired-end library was run on a MiSeq (Illumina).

Reads were quality trimmed to a minimum score of Q30 across the read. BWA (version 0.7.5) (11) was used to map 1,797,246 reads to the SEOV strain 80-39 reference genome (NCBI GenBank accession numbers NC_005238.1, NC_005237.1, and NC_005236.1), with 99.9% L, M, and S genome coverage, and the TCH S segment reference (NCBI GenBank accession no. AF329389), with 99.9% genome coverage. A consensus genome sequence was produced at a minimum depth of five reads. All analyses were performed using a local instance of the Galaxy Project (12 – 14). The previously reported TCH S segment contained an additional 16 bp close to the 3′ terminus that is not seen in the 3′ regions of other SEOV sequences (10). Mapping of the reads from this study to the TCH S segment reference revealed no support for the additional 16 bp.

### Nucleotide sequence accession numbers.

The whole-genome sequences have been deposited in GenBank under the accession numbers KU204958 to KU204960. This paper describes the second version of the sequences.
